# Systemic effects of oral tolerance improve the healing of several and concomitant wounds on different parts of the body

**DOI:** 10.1590/1414-431X2025e14689

**Published:** 2025-04-14

**Authors:** I.B.C. Nobrega, A.V.S. Andrade, T.J.N. Bikat, G.M. Quintão, G.M. Azevedo, K. Franco-Valência, R.A. Costa, C.R. Carvalho

**Affiliations:** 1 Departamento de Morfologia, Universidade Federal de Minas Gerais, Belo Horizonte, MG, Brasil; 2 Centro de Investigaciones en Ciencias de la Vida, Universidad Simón Bolívar, Barranquilla, Colombia; 3 Departamento de Ciências Naturais, Universidade Federal de São João del-Rei, São João del-Rei, MG, Brasil

**Keywords:** Wound healing, Immunological tolerance, Inflammation, Skin, External ear

## Abstract

Oral tolerance is an immunological phenomenon that results from protein intake and that has systemic effects on inflammation. Previous research has shown that parenteral injection of tolerated proteins reduces inflammatory infiltrate and improves skin wound healing. Herein, we tested whether the injection of tolerated proteins improves the healing of several wounds on different parts of the body, such as on the skin of the back and on the external ear (the auricle). To induce oral tolerance to ovalbumin (OVA), eight-week old C57BL/6 mice drank egg white diluted 1:5 in water for 3 consecutive days. The control mice drank water. Seven days after oral treatment, mice were submitted to excisional injuries on the skin of the back (6 mm) and ears (4 mm). Minutes before the injuries, the mice received an intraperitoneal injection of OVA + Al(OH)_3_. Seven and 40 days after the injuries, tissue samples were collected and processed for histological analysis of the wounds. The results showed that the injection of OVA in animals that drank OVA reduced the inflammatory infiltrate in all lesions. In addition, injection of OVA in animals that drank OVA promoted better organization of the extracellular matrix, with thicker and intertwined collagen fibers in the neodermis, resulting in smaller scars on the skin. Furthermore, the healing area of the ears of OVA-tolerant animals showed chondrocyte aggregates and less obvious fibrous scar tissue compared with control animals. In conclusion, systemic effects of oral tolerance positively influenced the healing of several lesions on different parts of the body.

## Introduction

Multiple injuries can occur in different situations including physical impact, chemical products, or pathological agents (1,2). In any situation, injuries result in disruption of the integrity of tissues and organs and, if not repaired in time, can have serious consequences for the body, including death. In humans, injuries are normally repaired by processes that replace the original tissue with a disordered extracellular matrix resulting in scars (3,4). Scars on the skin are easily visible as the closure of the lesions is normally not regenerative. Although dermal integrity is reestablished, it is replaced by scar tissue, a tissue morphologically and physiologically different from intact skin (5). In internal organs, scars can be noticed due to changes in their physiology.

Multiple injuries due to accidents or situations caused by our way of life or conflicts in our society are common and generate great demand for health services (1). Fortunately, there are many ways to treat wounds and save lives, but scars or delayed healing are still common. Therefore, studies are still needed to develop effective treatments for wound healing and scar reduction.

Inflammation is important at the beginning of repair and the resolution of inflammation is necessary for wound closure. Prolonged inflammation can result in chronic wounds or hypertrophic scars (5,6). Once changes in the inflammatory phase of repair influence subsequent phases, altering the inflammatory microenvironment with anti-inflammatory products and biomaterials is becoming an attractive approach in regenerative medicine (7).

It has long been known that one of the consequences of protein intake is oral tolerance, defined as refractoriness to subsequent immunization with the same protein (8,9). Parenteral injection of tolerated proteins has systemic effects that were shown to inhibit inflammation and immunization to other agents injected simultaneously, 3 days before or shortly after (10,11). Injection of ovalbumin (OVA) or zein plus adjuvant in animals fed with these proteins reduces the inflammatory infiltrate in lesions caused by different agents, whether physical (punch injury) (12,13), chemical (produced by carragenin) (14), or biological agents (*Schistosoma mansoni* eggs) (15,16). In cases of physical injuries, the positive effects of injecting tolerated proteins have been demonstrated in skin and bone injuries, resulting in improved wound healing (17).

The objective of this study was to verify whether the injection of a tolerated protein improves the healing of several injuries made at the same time and in different parts of the body of mice. Following ethical principles, injuries were made with a biopsy punch on the skin of the back and on the external ears of OVA-tolerant mice minutes after injecting OVA intraperitoneally (*ip*), and the wounds were analyzed 7 and 40 days after injuries.

## Material and Methods

### Animals

Male C57BL/6 mice, eight weeks old, were obtained from the Universidade Federal de Minas Gerais (UFMG), Brazil, and handled according to the guidelines of the Institutional Animal Care and Use Committee. The animals were divided into groups of 5 animals each for each time analyzed (n=5) and kept in a temperature-controlled environment at 24°C with a 12-h light/dark cycle.

### Treatment for induction of oral tolerance to ovalbumin (OVA)

**Figure 1:**
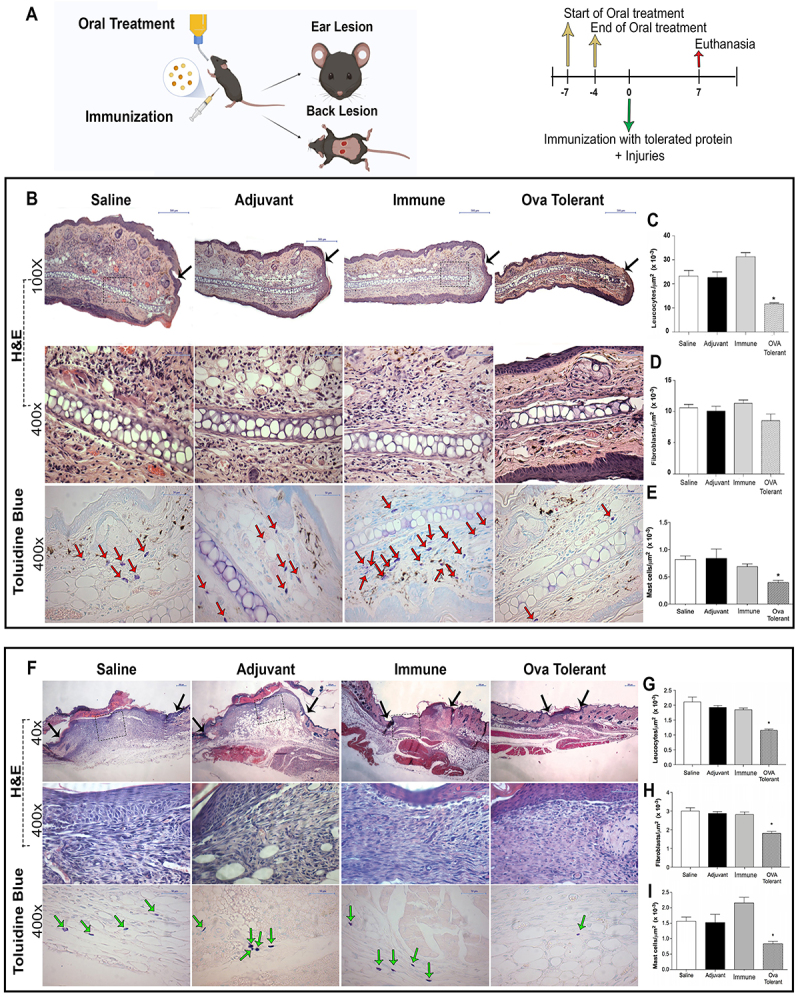
The *ip* injection of ovalbumin (OVA) in animals fed with this protein reduced the inflammatory infiltrate in the wound beds of the ear and back skin. **A**, Experimental design for analysis at day 7. **B**, Photomicrographs of ear sections 7 days post-injury, stained with H&E (black arrows indicate lesion border) or toluidine blue (red arrows indicate mast cells), scale bars: upper panels, 500 µm; middle and lower panels, 50 µm. Morphometry of (**C**) leukocytes, (**D**) fibroblasts, and (**E**) mast cells 7 days after ear lesions. **F**, Photomicrographs of the back skin 7 days post-injury stained with H&E (black arrows indicate lesion borders) and toluidine blue (green arrows indicate mast cells); scale bars: upper panels, 200 µm; middle and lower panels, 50 µm. Morphometry of (**G**) leukocytes, (**H**) fibroblasts, and (**I**) mast cells 7 days after lesions on the back skin. Data are reported as means±SE, n=5. *P≤0.05 compared with the saline group (ANOVA).

The experimental group received a solution of egg white diluted 1:5 in water (Figure 1A) as the only source of liquid for 3 consecutive days. The egg white solution was changed every day in the early evening. The control groups drank tap water.

### Intraperitoneal immunization and induction of systemic effects of oral tolerance

On the seventh day after oral treatment and 15 min before injuries, mice in the oral OVA-treated group (tolerant) and a control immune group received an *ip* injection of 10 μg of ovalbumin in 1.6 mg of Al(OH)_3_. A control group (adjuvant) received only the adjuvant Al(OH)_3_ and another group (saline) received saline.

### Injuries on the skin of the back and ears

Mice were anesthetized by *ip* injection of xylazine (16.5 mg/kg) and ketamine (97 mg/kg) diluted in saline and subjected to concomitant injuries on the skin of the back and ears. The skin on the dorsal region of the trunk was shaved and two excisional injuries were made using a 6-mm diameter surgical punch. An injury was made in the center of each ear with a 4-mm diameter surgical punch. Until recovery from anesthesia, the mice were kept blindfolded with cotton wool soaked in physiological saline to prevent corneal drying. After the injuries, the animals were kept in individual cages until the day of euthanasia.

### Preparation of samples for histological analysis

**Figure 2:**
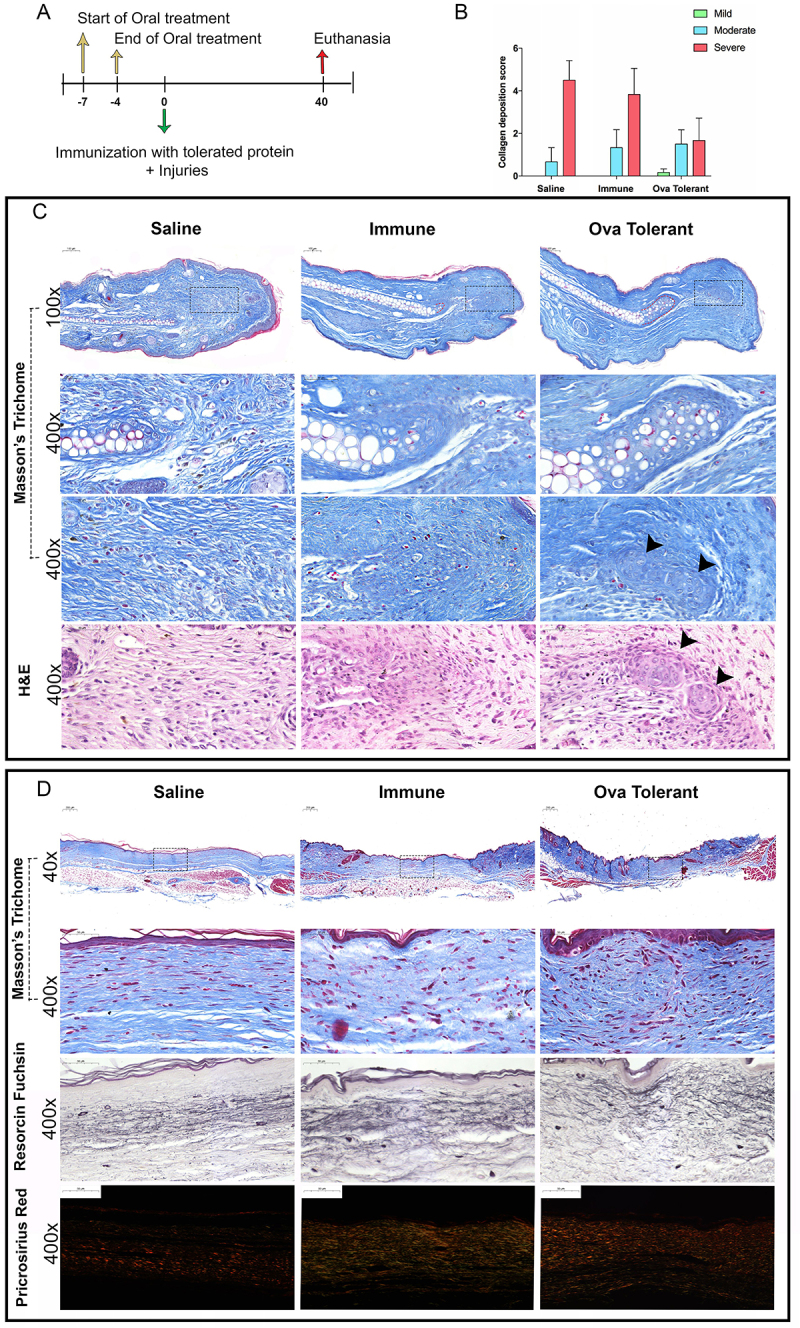
The *ip* injection of ovalbumin (OVA) in animals fed with this protein improved the healing of wounds on the ears and back skin. **A**, Experimental design for analysis at day 40. **B**, Collagen deposition score in samples analyzed 40 days after injuries on the skin of the back (n=5). **C**, Photomicrographs of the ear with the scar stained with Masson's trichrome or H&E. The red dotted line indicates chondrocytes formed at the site of cartilage injury. The area in the boxes was magnified (×400) to show chondrocyte aggregates (black arrows) and newly-formed chondrocytes extending from the original cartilage in OVA-tolerant mice. **D**, Photomicrographs of the back skin 40 days after injuries stained with Masson's trichrome to show collagen fibers, with Weigert's resorcin fuchsin to show elastic fibers, or picrosirius red to compare the thickness of collagen fibers.

At 7 and 40 days after the injuries (Figures 1A and 2A), the animals were anesthetized with xylazine and ketamine and euthanized by cervical dislocation. Tissue samples of the lesions were collected and fixed in Carlson formalin in Milloning buffer (pH 7.0) for 24 h and then transferred to 70% alcohol for at least 1 h before histological processing and inclusion in paraffin. The samples (n=5 per tissue, per time point) were sectioned at 5-µm thickness using a semi-automatic microtome (Microm HM 315, GMI, USA) and stained with hematoxylin and eosin (H&E), toluidine blue, Masson's trichrome, picrosirius red, and Weigert's resorcin fuchsin.

### Histological analysis

Histological sections were analyzed by two investigators in a double-blind manner using a light microscope (Olympus BX40, Japan) or a polarized light microscope (Olympus, BX43). The cells were counted by two investigators, in a double-blind manner, using an intersection grid attached to the ocular lens (Thomas Scientific, USA). Leukocytes, mast cells, and fibroblasts were identified by their characteristic color and morphology and counted in 10 fields of 10,000 μm^2^ each within the area of the lesions. Results are reported as means±SE.

### Scores of cutaneous scars

After microscopic analysis of the dorsal skin stained with Masson's trichrome at 40 days post-injury, we determined a score to compare the scars of the different groups. The scars were categorized as severe, moderate, or mild according to the intensity of deposition and organization of the collagen fibers. Scars were considered severe when the neodermis contained thinner, confluent collagen fibers parallel to the epidermis. In contrast, scars classified as mild showed thicker, intertwined, and closely packed collagen fibers, similar to the pattern observed in intact skin, but without epidermal attachments, such as hair follicles. Moderate scars presented a mixture of thin and thick collagen fibers. The scars were scored by two independent investigators and the results are reported in values for each category (1-2 for mild, 3-4 for moderate, and 5-6 for severe scar).

### Statistical analysis

Graphpad Prism software (USA) was used for statistical analyses. The significance of differences between groups was determined using one-way ANOVA followed by the Bonferroni test. Values of P≤0.05 were considered significant.

## Results

### Effects on the inflammatory infiltrate in wounds

Seven days after the injuries (Figure 1A), histological analyses of the wound beds on the ears of the animals that drank OVA showed a mild to moderate inflammatory infiltrate, with complete re-epithelialization and no cartilage at the injury site (Figure 1B). The saline, adjuvant, and immune control groups presented intense inflammatory infiltrate, hyperplastic epithelium, vascular congestion, and edema. The control groups also did not present cartilage at the injury site. Corroborating the qualitative analyses, morphometric analyses regarding the number of inflammatory cells in the wound beds of the ears showed a smaller number of leukocytes and mast cells in the group that drank OVA compared to the control groups (Figure 1C-E).

Regarding the wounds on the skin of the back, qualitative histological analyses at day 7 after the injuries showed that the group that drank OVA presented less inflammatory infiltrate and complete re-epithelialization, less vascularization, and less adipocytes in the wound bed compared to the control groups (Figure 1F). Morphometric analysis of the number of inflammatory cells and fibroblasts in the wounds on the skin of the back showed that the group that drank OVA had a lower number of leukocytes, mast cells, and fibroblasts compared to the control groups (Figure 1G-I).

### Effects on wound healing on the ears and back

At 40 days after ear injuries (Figure 2A), the animals that drank OVA showed a region of chondrocytes that had formed beyond the site of the cartilage injury, as highlighted by the red dotted line in the samples stained with Masson's trichrome (Figure 2C). This characteristic was not observed in the animals in the saline group, but was observed to a lesser extent in the immune group. Furthermore, the group that drank OVA presented less fibrous scar tissue compared to the control groups, in addition to the formation of chondrocyte aggregates in the region closest to the neoepithelium, visualized by Masson's trichrome and H&E staining.

At 40 days after the wounds on the skin of the back, histological analyses (Figure 2D) of the scars of the animals that ingested OVA revealed deposition of thick, intertwined collagen fibers, with few non-fibrous components of the extracellular matrix, similar to the uninjured skin. On the other hand, in the scars of the saline and immune control groups, collagen fibers were loosely organized, thinner, and parallel to the epidermis, typical characteristics of scar tissue. Furthermore, analysis performed after staining with picrosirius red, which allows a qualitative analysis of collagen fibers so that thicker collagen fibers appear in redder tones when analyzed under a polarized light microscope, showed that animals that drank OVA had a greater quantity of reddish fibers, a characteristic similar to that of uninjured skin (Figure 2D). On the other hand, control animals had a greater quantity of greenish fibers, a characteristic of thinner fibers normally found in scars. In addition, analysis of the elastic fibers 40 days after lesions on the skin of the back, using resorcin fuchsin staining (Figure 2D), showed that in the animals that drank OVA, but not in the control groups, there was a better reorganization of the elastic fibers, so that they appeared homogeneously distributed throughout the neodermis, a pattern similar to the elastic fibers found in uninjured skin.

The scar scores of the skin of the back were compared and showed that the scars in the control groups were predominantly severe, while the scars in the group that drank OVA were moderate or mild (Figure 2B).

## Discussion

The inflammatory infiltrate triggered by multiple wounds that were simultaneously made in different parts of the body was reduced by the injection of a protein that had been previously ingested. Furthermore, there was better reorganization of the extracellular matrix in the scars of the animals that received the injection of the protein after ingesting that protein. The absorption of proteins by the gut mucosa normally leads to the establishment of oral tolerance, a specific immunological phenomenon that inhibits immune responses to the ingested proteins even when they are injected together with adjuvants (18). Previous work has shown that the injection of tolerated proteins does not result in immune responses rather produces systemic and anti-inflammatory effects (19). Other research has already shown that the systemic effects of oral tolerance, triggered by intraperitoneal or subcutaneous injection of dietary proteins, modify the initial phases of skin repair and reduce scars (20,21). This is the first work to show that the systemic effects of oral tolerance can improve the healing of concomitant wounds made in different parts of the body.

One of the characteristics of wound repair modified by the systemic effects of oral tolerance was the reduction in the number of mast cells in the lesion area, as shown in Figure 1. Mast cells can release a large number of growth factors and cytokines and the action of these cells during wound repair may involve multiple mechanisms (22). Mast cells are involved in all phases of repair, from the initial inflammatory phase to collagen deposition, by influencing the activity of fibroblasts. Different studies have shown an increase in the number of mast cells in hypertrophic scars (22). Other studies have shown that a decrease in mast cells in the injured area correlated with better healing (23). During the inflammatory phase, factors produced by mast cells promote the recruitment of neutrophils and macrophages, and blocking mast cell activation in the early phase of repair reduces skin scarring (24). Furthermore, mast cells may interact directly with fibroblasts, promoting their activation and the production and remodeling of the extracellular matrix (25).

Studies have shown that reducing the inflammatory phase of wound repair correlates with reduced scarring and regeneration (5). Our results showed a reduction in the number of inflammatory cells 7 days after the injuries and better reorganization of collagen fibers and elastic fibers 40 days after the injuries on the skin of the back. Furthermore, there was a reduction in the number of inflammatory cells in the injuries on the external ear (auricle) that resulted in a reduced fibrotic repair, including the formation of chondrocyte aggregates. The presence of chondrocyte aggregates suggested that the systemic effects of the injection of tolerated proteins may benefit the regeneration of a tissue that normally does not present a good regeneration potential (such as cartilage). These results, together with previously published results showing a positive effect of the injection of tolerated proteins in the repair of bone defects (17), suggest a great potential for the use of this technique in the treatment of osteochondral defects. Further studies need to be conducted to translate the results obtained with animal models to the clinic.

Reduced fibrotic repair may result from the reduction of the inflammatory infiltrate itself, since the actions of inflammatory cells can produce lesions in the surrounding tissue (26). Furthermore, increased inflammatory infiltrate may delay the resolution of inflammation and hinder the reorganization of damaged tissue (27). However, our results do not exclude the action of the systemic effects of oral tolerance from the injection of tolerated proteins on the migration and activation of fibroblasts.

Although we did not study the specific mechanisms triggered by the injection of the tolerated protein, previous works have shown that the systemic effects of oral tolerance by injection of the ingested protein produce changes in the expression of adhesion molecules and in the kinetics of cytokines, which may explain the reduction of the inflammatory infiltrate in the wound bed (15,28).

Previous studies have shown that *ip* injection of a protein previously given orally concomitantly with intravenous injection of *Schistosoma mansoni* eggs reduces the formation of pulmonary granulomas (16). A recently published study showed that *ip* injection of a protein present in the rat diet reduced the inflammatory infiltrate in a bone fracture and promoted better bone repair (17). The evidence that the systemic effects of oral tolerance can reduce the inflammatory infiltrate in lesions of different organs and thus reduce fibrosis suggests a significant clinical application in cases of multiple injuries, such as surgeries, traffic accidents, falls, or sports injuries (1,29). However, the technique must be first tested in humans.

## Conclusions

Oral administration of ovalbumin followed by the injection of this protein concomitantly with several wounds made in different parts of the body resulted in a reduction in the inflammatory infiltrate and improved wound healing, favoring the formation of less pronounced scars.
